# Soil respiration variation along an altitudinal gradient in the Italian Alps: Disentangling forest structure and temperature effects

**DOI:** 10.1371/journal.pone.0247893

**Published:** 2021-08-17

**Authors:** Aysan Badraghi, Maurizio Ventura, Andrea Polo, Luigimaria Borruso, Francesco Giammarchi, Leonardo Montagnani

**Affiliations:** 1 Faculty of Science and Technology, Free University of Bozen-Bolzano, Bolzano, Italy; 2 Forest Services, Autonomous Province of Bolzano, Bolzano, Italy; Tennessee State University, UNITED STATES

## Abstract

On the mountains, along an elevation gradient, we generally observe an ample variation in temperature, with the associated difference in vegetation structure and composition and soil properties. With the aim of quantifying the relative importance of temperature, vegetation and edaphic properties on soil respiration (SR), we investigated changes in SR along an elevation gradient (404 to 2101 m a.s.l) in the southern slopes of the Alps in Northern Italy. We also analysed soil physicochemical properties, including soil organic carbon (SOC) and nitrogen (N) stocks, fine root C and N, litter C and N, soil bulk densities and soil pH at five forest sites, and also stand structural properties, including vegetation height, age and basal area. Our results indicated that SR rates increased with temperature in all sites, and 55–76% of SR variability was explained by temperature. Annual cumulative SR, ranging between 0.65–1.40 kg C m^-2^ yr^-1^, decreased along the elevation gradient, while temperature sensitivity (Q10) of SR increased with elevation. However, a high SR rate (1.27 kg C m^-2^ yr^-1^) and low Q10 were recorded in the mature conifer forest stand at 1731 m a.s.l., characterized by an uneven-aged structure and high dominant tree height, resulting in a nonlinear relationship between elevation and temperature. Reference SR at 10°C (SR_ref_) was unrelated to elevation, but was related to tree height. A significant negative linear relationship was found between bulk density and elevation. Conversely, SOC, root C and N stock, pH, and litter mass were best fitted by nonlinear relationships with elevation. However, these parameters were not significantly correlated with SR when the effect of temperature was removed (SR_ref_). These results demonstrate that the main factor affecting SR in forest ecosystems along this Alpine elevation gradient is temperature, but its regulating role can be strongly influenced by site biological characteristics, particularly vegetation type and structure, affecting litter quality and microclimate. This study also confirms that high elevation sites are rich in SOC and more sensitive to climate change, being prone to high C losses as CO_2_. Furthermore, our data indicate a positive relationship between Q10 and dominant tree height, suggesting that mature forest ecosystems characterized by an uneven-age structure, high SR_ref_ and moderate Q10, may be more resilient.

## Introduction

Soil respiration (SR) is the largest biological carbon (C) flux after photosynthesis in terrestrial ecosystems, and is estimated to release 50–77 Pg C yr^-1^ globally [1–3]. This major natural flux largely determines the C balance between the terrestrial biosphere and the atmosphere [4–6] and plays a critical role in the carbon cycle. Soil is the largest C pool in the terrestrial biosphere and has been increasingly recognized to play a crucial role in mitigating global warming resulting from climate change [7–9]. Small changes in soil CO_2_ efflux or soil organic C stocks could severely impact the global C cycle [10]. The important role of forest productivity in the determination of SR was first demonstrated by Janssens et al. [11] and other studies have subsequently investigated and quantified the impact of productivity on SR modeling [12, 13]. In addition to productivity, SR is influenced by different abiotic and biotic factors such as soil temperature, soil moisture, and microbial community composition, introducing considerable uncertainty in SR estimates [14–16]. Among these factors affecting respiratory processes, the temperature has been the most well-studied [17, 18]. Many studies have addressed the prediction of SR response to increasing temperature (i.e. temperature sensitivity of SR), producing different equations relating soil CO_2_ efflux to temperature [19–21] or a combination of temperature and soil humidity [22]. However, the Q10 function [23], using the Q10 parameter to describe the temperature sensitivity of SR, is one of the most widely used models to quantify CO_2_ efflux from the soil in Earth system models.

The elevation is a key driver of climate properties, playing an essential role in soil organic matter content and mitigating the effects of climate change [16, 24–27]. Typically, temperature declines with elevation, thus elevation gradients have been widely used to assess soil respiration response to temperature. Studies suggest that CO_2_ exchange between soil and atmosphere varies along climatic gradients and that the temperature sensitivity (Q10) of SR increases with elevation [14, 28–30]. Furthermore, a positive relationship between soil organic carbon (SOC) and elevation indicates that global soil organic C stocks at high elevation are more sensitive to climate change and are predicted to decrease in a warming climate [16, 25, 31–34]. However, other studies have reported opposite trends, finding lower SOC content and higher SR at high elevations [32, 35, 36]. This variability may be partially due to confounding factors, other than temperature, affecting SR. Besides elevation, mountain landscapes are, in fact, characterized by substantial local changes in other parameters such as slope angle and orientation, which can affect microclimatic conditions and, therefore, soil C dynamics [37]. Furthermore, due to the heterogeneity of geological substrates, soils in mountain regions show high small-scale heterogeneity, which can generate marked differences in soil biogeochemical properties [38]. Studies have also found conflicting results in terms of the relationship between SR and SOC [28, 39].

Furthermore, the various plant communities can affect soil respiration rate through the diverse microclimate, soil and litter composition, and root distribution [20, 28, 40–42]. However, within the same plant biome, there is also high spatial heterogeneity in SR. Some authors have found a possible linkage between the topography, plant community structure (e.g., forest type and speed of regeneration), and SR within the same forest ecosystem [20, 40–42]. Furthermore, forest management can also play a crucial role in SR [43]. For example, tree removal can directly influence SR due to the removal itself (i.e., reduction of plant biomass) but also through indirectly changing the soil physicochemical properties and micrometeorological conditions [44].

Currently, the temperature dependency of SR and SOC decomposition is of major interest in the study of global climate change and the role of terrestrial ecosystems in regulating Earth´s climate [45, 46]. Therefore, there is a need to better understand the interactions between temperature and soil CO_2_ efflux. The general goal of this study was to disentangle the possible multi-effects of soil properties, temperature, SOC, and vegetation structure (in particular tree height and age) on SR along a plant biome elevation gradient. In particular, the existing differences in vegetation structure allowed us to investigate the extent to which these biological variables can alter the relation between elevation and SR.

Specifically, we tested the hypotheses that i) SR and SOC accumulation change linearly with elevation; and ii) the Q10 value increases linearly with elevation. Furthermore, we determined the main factors, other than temperature, affecting SR. To effectively isolate the effect of temperature on SR, the study was conducted along an altitudinal gradient in the Italian Alps, where confounding factors such as slope angle and orientation, and soil parent material were minimized. The differences in vegetation structure allowed us to investigate to which extent these biological variables, and the induced variation in microclimatology, can alter the relationship between elevation and SR.

## Material and methods

### Study sites

**Fig 1:**
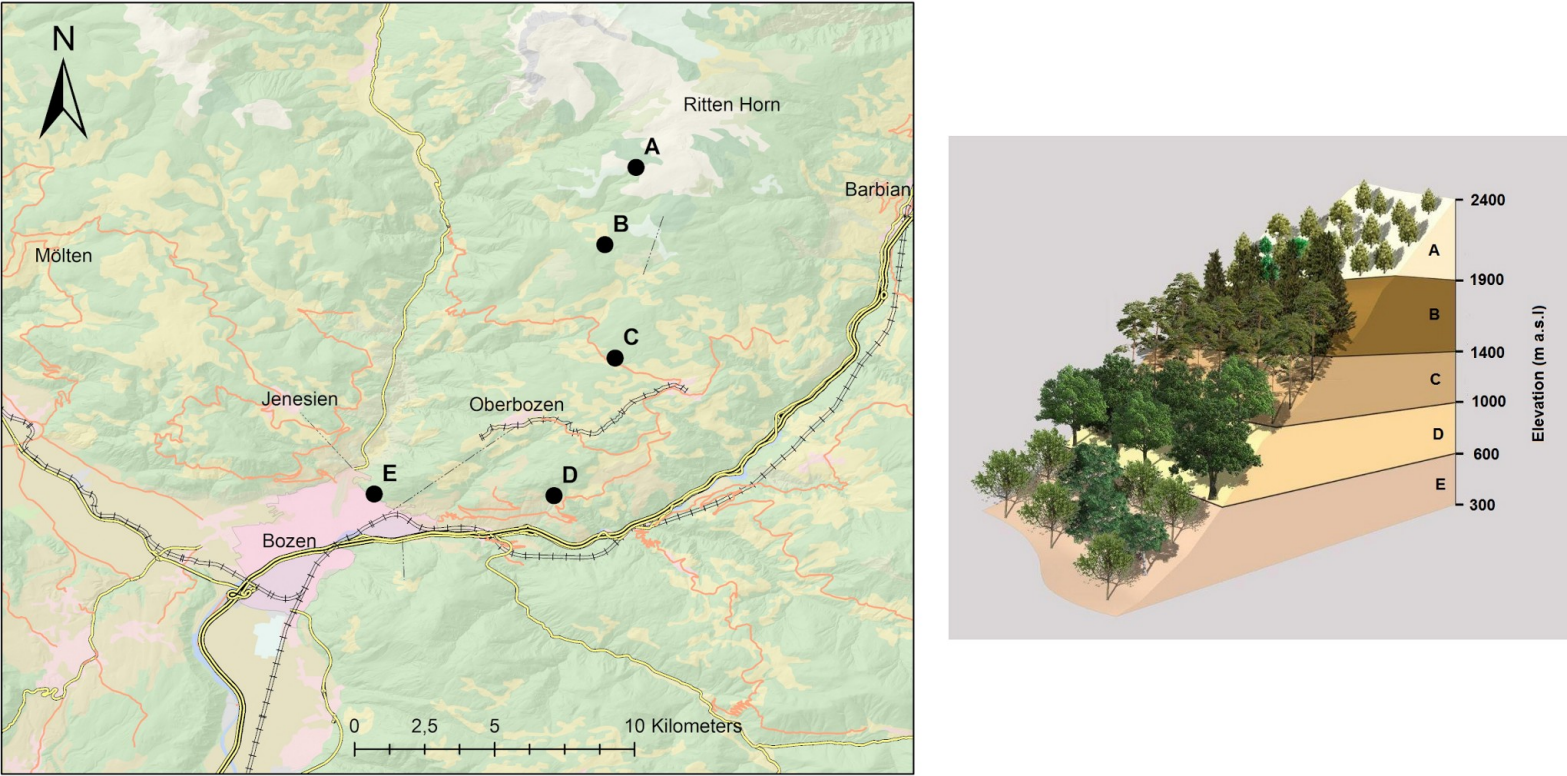
(a) Map showing study sites (b) Scheme of vegetation along the elevation gradient.

Five experimental sites were established between the top of the Rittner Horn and the city of Bozen, Italy, on the southern side of the Alps (Fig 1A). The overall elevation gradient between the highest (A) and the lowest site (E) is 1697 m and the elevation separation between each site is approximately 424 ± 60 m. All sites are characterized by the soil developed upon a glacial till laid on a porphyric bedrock and a SE slope orientation. Previous analyses performed at site B revealed that the soil was poor in ions forming carbonates (Ca^++^ = 106.2 meq/kg, Mg^+^ = 58.0 meq/kg), indicating that the spatial variability of soil C is largely determined by SOC. Soil CO_2_ flux measurements were taken in 2017 and 2018, during which time annual average precipitation ranged between 746 mm (Bozen, near the lowest site (site E)) and 1090 mm (measured near-site B at 1780 m) (data from the Hydrographic Office and the Agency for the Environment of the Autonomous Province of Bozen). Based on Wehren et al. [47], precipitation was expected to increase along the elevation gradient, with a minimum at site E and a maximum at site A. Details on the main characteristics of the research sites are reported in Table 1 and S1 File.

**Table 1:** General characteristics of the study sites.

Characteristics	Site A	Site B	Site C	Site D	Site E
Elevation (m a.s.l.)	2101	1731	1354	865	404
Mean annual temperature (°C)	4	4	12	11	14
Slope orientation	SE	SE	SE	SE	SE
Slope inclination (%)	15	18	12	18	25
Stand age (yr)	61	202	153	54	88
Land use	Shrubland	Forest	Forest	Forest	Forest
Dominant tree height (m)	1.9	29	22.5	18.8	8.7
Basal area (m^2^ ha^-1^)	7.1	42.0	42.7	39.8	22.8
Dominant overstory species	Dwarf Mountain pines (*Pinus mugo*)	Norway spruce (*Picea abies*), Swiss stone pine (*Pinus cembra)*, Larch (*Larix decidua*)	Scotts pine (*Pinus sylvestris*)	Sessile oak *(Quercus petrea)*, Scotts Pine *(Pinus sylvestris)*, Chestnut (*Castanea sativa*)	Sessile oak *(Quercus petrea)*, Flowering ash (*Fraxinus ornus*)
Main understory species	Intervening grasses (*Festuca halleri*)	Rusty leaved alprose (*Rhododendron ferrugineum*)	Heather (*Erica carnea*)	Understory almost absent	Smoke-bush (*Cotinus coggygria*), Succulent plants (*Opuntia humifusa*), Scorpion senna (*Hippocrepis emeru*s).

Site A was established in even-aged shrubland vegetation composed of Dwarf Mountain pine (*Pinus mugo* Turra) near the summit of the Rittner Horn. Pine trees at site A showed an even-aged distribution based on trunk diameter. The mean age was 61 yr.

Site B was established in a subalpine Norway spruce stand (*Picea abies* (L.) Karst.) at the Fluxnet research station of Renon-Mittelgrünwald (http://sites.fluxdata.org/IT-Ren/). The site is characterized by an uneven-aged distribution of individuals based on tree diameter, approaching the structure of old-growth forest stands [48, 49]. This type of structure was achieved by a traditional sylviculture treatment based on cutting small groups of trees. The oldest group of trees had a mean age of 202 yr.

Site C was located near Riggermoos, in a Scotts pine (*Pinus sylvestris* L:) stand, displaying a large basal area and an even-aged structure (mean age 153 yr.).

Site D was established in a mixed stand of Sessile oak (*Quercus petrea* (Matt.) Liebl.) and Chestnut (*Castanea sativa* L.), with some individuals of Scots pine and larch (*Larix decidua* L.) near the village of Signat. Forest vegetation at this site evolved from a former oak-chestnut coppice, with the presence of individuals of pines and larches grown as standards (mean suckers age 54 yr.).

Site E was located in a low stature stand dominated by Sessile oak and Flowering ash (*Fraxinus ornus* L.) on the hill slope of Sankt Magdalena, close to Bozen, with the entry in the plant community of tree species cultivated in the town gardens, for example, Lawson cypress (*Chamaecyparis lawsoniana* (A. Murray) Parl.

All sites except sites A and E are managed as high forests, mainly for wood harvesting. Site A is managed as natural vegetation with occasional harvesting at the forest margins to avoid expansion into the adjacent pasture areas. Site E has not been managed for decades. Tree age was assessed during sampling in 2018 and 2021 based on tree ring count. Forest tree diameter and height in circular plots of variable radius (5–25 m) were assessed during two sampling campaigns (2020 and 2021) with a TruePulse 360 B (Laser Tech, CO, USA).

The permission to carry out the experiment was obtained through the Forest Services of the Autonomous Province of Bozen (in case of public land, sites A and E) or by signed agreement with the landowners, in case of private land (sites B, C and D).

### Soil respiration

To quantify SR, ten iron collars (10 cm height, 20 cm diameter) were inserted into the soil, three weeks before the first measurements at each site. Measurements were performed with an opaque survey chamber (Li-8100-104, LI-COR Biosciences, NE, USA) connected to an LI-8100 analyzer (LI-COR). By using this technique, an integrated value of SR is provided, which includes the heterotrophic CO_2_ emission from the different soil horizons and from the litter and the autotrophic CO_2_ emission from roots. On each collar, the measurement period was set to 120 s; the first 20 s of the measurement were considered dead-band, the last 20 s of purging, so the flux computation was limited to 80 s (see Montagnani et al. [10] for further details concerning the measurement settings). From July 21, 2017, SR measurements were performed periodically, about once per month, until July 20, 2018, for a total of 17 measurement days. The first four series of measurements were performed every three weeks at all the sites. During the winter, SR measurements were performed only in the lower elevation sites due to snow at higher elevations. The measurement calendar and flux data for the different sites are provided in S2 File. During measurements, the air temperature was measured inside the survey chamber (at 0.1 m above ground, RHT Plus, Skye Instruments, UK). A soil temperature profile was installed at control site B according to ICOS protocol [50]. Specifically, we used the -5 cm soil T data provided by a CS605 probe (Campbell Scientific, USA). We recorded soil temperature continuously at 30 min intervals throughout the experimental period.

Soil respiration data collected from each measurement point (collar) were related to chamber air temperature using a logistic model [28]:

$$SR=\frac{a}{\left(1+b\cdot \exp(-k\cdot T_{a}\right)}$$ Eq 1

Where SR is soil respiration, a is the maximum value of SR, b determines the elongation of the SR curve along the x-axis, k is the logistic growth rate or steepness of the SR curve along the x-axis, and T_a_ is air temperature. Furthermore, SR data were also fitted with a Q10 model [51, 52]:

$$SR=SR_{ref}\cdot Q_{10}{}^{\left(\frac{\left(Ta-Tref\right)}{10}\right)}$$ Eq 2

Where SR is the soil respiration, SR_ref_ is the fitted SR at the reference temperature of 10°C (T_ref_), Q10 is the temperature sensitivity of SR, defined as the factor by which soil respiration increases with a 10°C temperature increase, and Ta is chamber air temperature.

### Soil and plant analysis

After taking the last measurements (July 2018), the soil in each collar was sampled to a depth of 20 cm using a 4.8 split-corer (Eijkelkamp, NL). Litter present in each collar, which included leaf, cones and branch fragments was collected separately from the soil. Fine roots (<2 mm diameter) were separated from coarse roots using a caliper and were weighed after oven-drying at 105 ± 5°C. Larger roots (>2 mm) were analysed together with the soil. By using this fixed-depth approach of soil sampling, different soil horizons can be included in the sample. Being the soil organic (O horizon) depth different at the different locations, at site B, the O horizon only was included in the samples, while at the other sites the top of the mineral horizon was included as well, at least in a subset of samples.

In the laboratory, soil samples were weighed and sieved (2 mm mesh size) to separate roots, stones, and coarse organic matter fragments. The stones removed from each sample were weighed and their volume was determined on the base of their density. Soil bulk density was determined by dividing the weight of sieved soil by the sample volume, which was calculated substracting the volume of the stones from the core volume. Soil pH was measured using a pH-meter (Crison pH-Meter Basic 20+Electrod: Hach 50 10T CRISON, Barcelona, Spain) in a suspension with a soil:water ratio of 1:5. Fine roots, litter, and soil samples were analyzed for SOC and N content using a FlashEA^TM^ 1112 Elemental Analyzer (Thermo Fisher Scientific, Waltham, MA, USA).

Soil organic C stock was obtained as follows [53]:

$$Soil\;stock\;(kg\;dm^{-3})=C/100\times BD_{s}{}_{oil}\;(kg\;m^{-3})\times 0.2\;(m)$$ Eq 3

where C is the SOC content, BD_*soil*_ is the soil bulk density (kg dm^−3^) and 0.2 m is the sampling depth. Soil N stock was calculated with the same formula, using total N content in the place of SOC content. Since all sites were established on acidic soil and porphyric bedrock, carbonates contribution to total soil C were negligible and total soil C was almost equivalent to SOC.

Fine root C and N stocks were determined with the same computational approach, using fine root density (kg dm^-3^) instead of soil BD and fine root C and N content instead of SOC and N content. Litter C and N amounts were obtained by multiplying the litter dry weight by C or N content and dividing by the collar area. Data concerning soil and plant analysis is reported in S3 File.

In order to estimate the stand age at each site, three dominant trees of the most common species were cored with a 5 mm increment borer, then partially planed with a microtome blade to reveal growth rings. Lastly, age was measured through the LINTAB measuring device linked to the TSAP-Win software (Rinntech, Germany), following standard dendrochronological procedures [54].

### Data analysis

Models were fitted to SR data using the nls package in R software [55]. Model fitness was evaluated based on Akaike’s Information Criterion (AIC), R-squared (R^2^), Mean Absolute Error (MAE), and Root Mean Squared Error (RMSE). The Q10 model was used to obtain the SR_ref_ and the Q10 value for every collar. Linear regression was used to compare the air temperature measured continuously in the reference plot (site B) and air temperature inside the chamber during SR measurements for each collar in each site. The obtained linear regression models were then used to predict chamber air temperature for the whole experimental period, for each collar, with a 30 min resolution (S4 File). Therefore, the predicted chamber air temperature was used to predict SR values simultaneously for the whole experimental period, based on the logistic models relating SR to chamber temperature. For some collars, it was not possible to obtain a good fit of the SR data using the logistic model; for these collars, the predicted soil respiration data from temperature was obtained using the Q10 model developed for the same collar. Finally, the total cumulative SR for the whole experimental period was determined for each collar at each site.

Soil respiration response to biological variables (SOC content, fine root C, litter C, fine root dry weight, soil N, fine root N, litter N, litter dry weight) was examined using Spearman’s Correlation Test and linear mixed-effects models (LMMs) fitted by restricted maximum likelihood (REML). Before applying LMMs, to avoid statistical errors, Variance Inflation Factor (VIF) was determined for biological variables, and variables with high VIF values were excluded from the model assessment. LMMs were built using the lme4 R package [56–58]. The models consisted of both fixed and random effects: biological variables were considered as fixed effects, and sampling plots (collars) nested in each site were used in the random-effects formula. R^2^ was used to summarize model goodness-of-fit together with AIC [59, 60]. Since computed R^2^ values by LMMs are a pseudo-R^2^ and technically incorrect, the r2glmm R package was used to compute R^2^ [60]. To exclude the confounding effect of temperature from LMMs and correlations tests, environmental variables were related to SR_ref_ instead of SR [22, 28, 61]. Furthermore, to assess the correlation between gross primary production (GPP) and SR, dominant tree height was used as a covariate in LMMs and Spearman’s correlation test. Statistical comparisons of average SOC and N content, fine root C and N, litter C and N, soil bulk density, soil pH, and soil respiration in the different sites were performed using the Kruskal-Wallis test (Dunn test, p < 0.05) for the non-normally distributed data, and a one-way ANOVA for the normally distributed data (Tukey test, p < 0.05). The normality of the data and homogeneity of variance were checked by the Shapiro–Wilk test and Levene’s test, respectively [62, 63]. To test linearity changes of SR, Q10 and SOC content with elevation, linear and nonlinear polynomial regressions were applied between elevation and environmental variables (SOC and soil N content, fine root C and N, litter C and N, soil bulk density, soil pH). The linearity changes of these variables with elevation were detected based on the lowest AIC and the highest R^2^. The association of Q10, SOC and soil N content with environmental variables was determined using Spearman’s Correlation Test. All statistical analyses were performed using R version 3.6.0 ([55], www.r-project.org).

## Results

### Environmental factors variability along the altitudinal gradient

**Fig 2:**
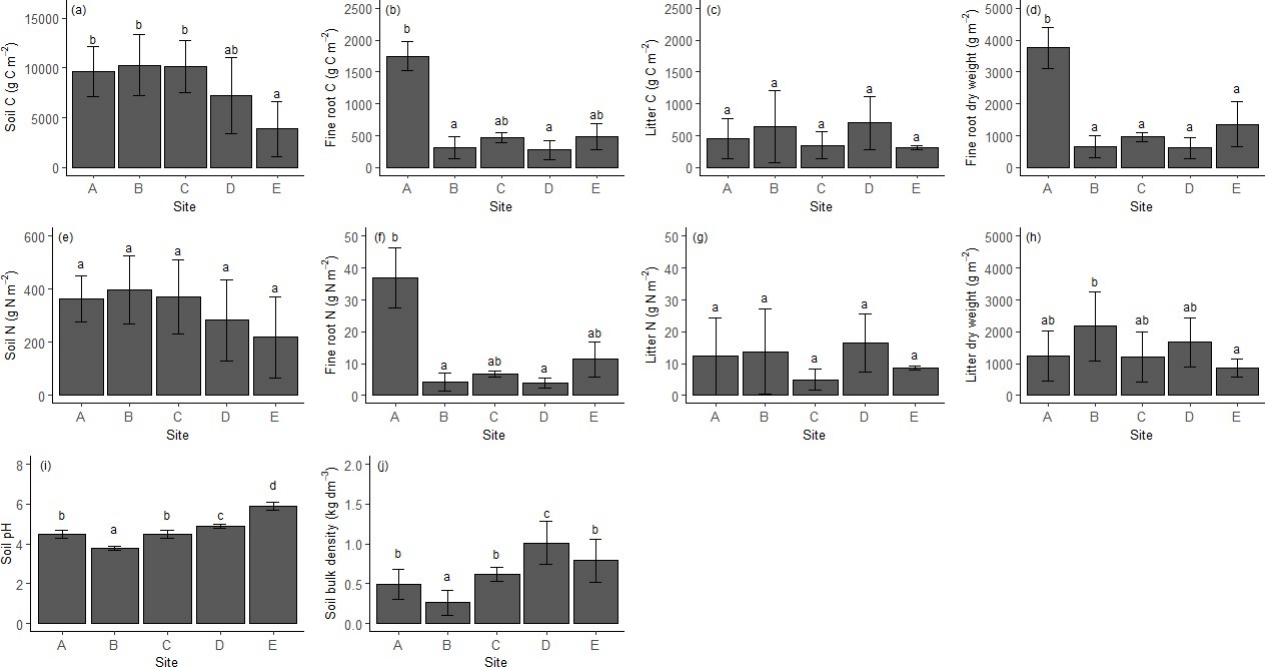
Stocks of C and N in soil and biomass (a-g); litter dry weight (h); soil pH (i); and soil bulk density (j), in the examined sites (A-E). Different letters indicate significant differences between sites. Vertical bars represent the standard deviation of the mean.

A significant difference in SOC stock was found only between site E (3891 ± 2756 g C m^-2^), at the lowest elevation where the C stock was smallest, and sites A, B, and C (Fig 2A). No significant differences were found in soil N stock between the different sites (Fig 2E). Fine root biomass and fine root C and N stocks in site A were significantly higher than in other sites (Fig 2B, 2D and 2F) and litter dry weight in site B was significantly higher than site E (Fig 2H). However, the accumulated C and N in the litter did not significantly differ along the altitudinal gradient (Fig 2C and 2G). Significant differences were found between pH values in the different sites (Fig 2I): the lowest soil pH was measured in site B (pH 3.8 ± 0.1) and the highest in site E (pH 5.9 ± 0.2, Fig 2I). The highest bulk density was found in site D (1.01 ± 0.27 g cm^-3^, Fig 2J) and the lowest was found in site B (0.26 ± 0.16 g cm^-3^; Fig 2J).

Based on the AIC and R^2^, only soil bulk density was linearly related to elevation (Table 2). On the contrary, SOC and soil N stock, fine root dry weight, fine root C and N stock, pH and litter dry weight were better fitted with nonlinear relationships with elevation (Table 2; further detail about equation parameters can be found in S5 File). Furthermore, a significant correlation was found between SOC and soil N and soil pH, dominant tree height, stand age, basal area, and bulk density (Table 3).

**Table 2:** Linear (lm) and polynomial regressions (poly) between the different parameters tested and elevation.

Parameters	Elevation					
	Significance		Multiple R^2^		AIC	
	lm	Poly	lm	Poly-lm	lm	Poly-lm
SOC stock (kg C m^-2^)	***	***	0.28	**0.40**	764	**758**
Soil N stock (g N m^-2^)	*	*	0.15	**0.20**	510	**509**
Soil respiration (kg C m^-2^ yr^-1^)	***	***	0.33	**0.36**	34	34
Temperature sensitivity of soil respiration (Q10)	***	**	0.28	**0.41**	77	**67**
Respiration at the 10°C reference temperature (SR_ref_) (kg C m^-2^ yr^-1^)	n.s.	n.s.	<0.01	0.08	139	139
Fine root dry weight (kg m^-2^)	*	**	0.10	**0.24**	717	**712**
Fine root C stock (kg C m^-2^)	*	***	0.39	**0.80**	199	**186**
Fine root N stock (g N m^-2^)	n.s.	***	0.28	**0.80**	102	**88**
Litter dry weight stock (g m^-2^)	n.s.	*	0.07	**0.12**	774	**772**
Litter C stock (kg C m^-2^)	n.s.	n.s.	< 0.01	0.04	222	223
Litter N stock (g N m^-2^)	n.s.	n.s.	< 0.01	< 0.01	112	114
pH	**	***	0.63	**0.88**	22	**8**
Soil bulk density (kg dm^-3^)	***	***	**0.32**	0.29	**9**	11

*—p ≤ 0.05;

**- p ≤ 0.01;

***—p ≤ 0.001; n.s.–not significant. BD–soil bulk density; Q10 –temperature sensitivity of soil respiration; SR -–cumulative soil respiration; SRref–respiration at the 10°C reference temperature; AIC–Akaike information criterion.

**Table 3:** Spearman rank coefficients for the correlations between different variables.

Variable	SOC stock (kg C m^-2^)	Soil N stock (g N m^-2^)	Temperature sensitivity of soil respiration (Q10)
SOC stock (kg C m^-2^)	1.00	0.94***	0.15^n.s.^
Soil N stock (g N m^-2^)	0.94***	1.00	0.13^n.s.^
Fine root dry weight (g m^-2^)	0.14^n.s.^	0.11^n.s.^	0.06^n.s.^
Fine root C stock (kg m^-2^)	-0.11^n.s.^	0.36^n.s.^	0.37^n.s.^
Fine root N stock (g N m^-2^)	-0.07^n.s.^	0.32^n.s.^	0.46^n.s.^
Litter dry weight (kg m^-2^)	0.02^n.s.^	0.01^n.s.^	-0.08^n.s.^
Litter C stock (kg C m^-2^)	-0.17^n.s.^	-0.36^n.s.^	0.01^n.s.^
Litter N stock (g N m^-2^)	-0.09^n.s.^	-0.23^n.s.^	-0.44^n.s.^
pH	-0.62*	-0.54*	-0.29^n.s.^
Soil bulk density (kg dm^-3^)	-0.52*	-0.39*	-0.23^n.s.^
Dominant tree height (m)	0.33*	0.21^n.s.^	-0.29*
Basal area (m^2^)	0.31*	0.16^n.s.^	-0.27*
Stand age (yr)	0.32*	0.26^n.s.^	-0.11^n.s.^

Asterisks indicate significance levels: *–p ≤ 0.05,

**–p ≤ 0.01, and

***—p ≤ 0.001; n.s.–nonsignificant. SOC–soil organic carbon.

### Soil respiration

**Fig 3:**
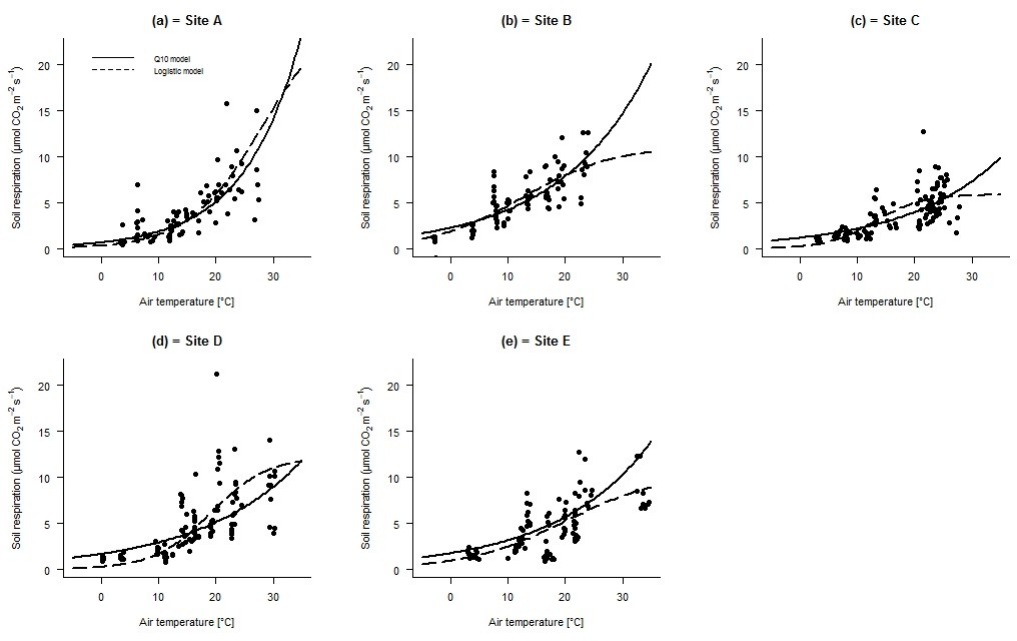
Rates of soil respiration against chamber air temperature in the different sites. Regression lines were built using the mean values of model parameters obtained for different replicate collars of each site (n = 10).

Both logistic and Q10 models confirmed that soil respiration rates increased with temperature in all sites (Fig 3), and the seasonal pattern of SR was similar to that of air chamber temperature (S6 File).

A strong linear relationship was found between observed and predicted SR (R^2^ = 0.73; S7 File). Temperature explained between 55% and 76% of the variance in soil respiration at the experimental sites (Table 4). The Q10 and SR_ref_ values obtained for the different sites ranged between 1.75 and 2.96, and between 2.17 and 4.49 (μmol CO_2_ m^–2^ s^–1^), respectively (Table 4). The Q10 value recorded in site A (highest elevation) was significantly different from that of the other sites (Table 4). A significant linear relationship was identified between Q10 and elevation (Table 2). However, the trend between Q10 and temperature was best described by a nonlinear relationship (Table 2). Furthermore, a significant negative correlation was found between Q10 and mean dominant tree height and basal area (Table 3). No significant relationship between SR_ref_ and elevation was found (Table 2). However, significant differences were found between experimental sites; the highest SR_ref_ value was recorded in site B and the lowest values in sites A, C, and E (Table 4).

**Table 4:** Mean values of Q10 (temperature sensitivity) and SR_ref_ (soil respiration at the temperature of 10°C) for each site (A, B, C, D and E).

	A	B	C	D	E
Q10	2.96 ± 0.72^b^	1.83 ± 0.25^a^	1.90 ± 0.24^a^	1.75 ± 0.15^a^	1.75 ± 0.32^a^
SR_ref_ (kg C m^-2^ yr^-1^)	2.17 ± 0.60^a^	4.49 ± 0.71^c^	2.24 ± 0.58^a^	3.17 ± 1.03^b^	2.98 ± 0.41^ab^
AIC logistic model	32.95 ± 10.45	34. 25 ± 6.45	34.19 ± 11.65	48.00 ± 14.55	49.76 ± 6.70
AIC Q10 model	30.42 ± 13.69	34.07 ± 7.31	35.39 ± 11.53	48.44 ± 14.13	49.11 ± 6.60
R^2^ logistic model	0.76 ± 021	0.75 ± 0.20	0.75 ± 0.16	0.67 ± 0.11	0.58 ± 0.10
R^2^ Q10 model	0.75 ± .23	0.71 ± 0.23	0.68 ± 0.21	0.61 ± 0.11	0.55 ± 0.11
MAE logistic model	1.06 ± 0.65	1.06 ± 0.45	0.68 ± 0.32	1.26 ± 0.62	1.29 ± 0.25
MAE Q10 model	1.07 ± 0.68	1.14 ± 0.52	0.79 ± 0.36	1.46 ± 0.76	1.39 ± 0.26
RMSE logistic model	1.36 ± 0.94	1.27 ± 0.50	0.86 ± 0.44	1.70 ± 0.89	1.71 ± 0.36
RMSE Q10 model	1.38 ± 1.0	1.36 ± 0.55	0.99 ± 0.49	1.87 ± 1.02	1.77 ± 0.38

MAE (Mean Absolute Error), RMSE (Root Mean Squared Error); R2 (Determination Coefficient) and AIC (Akaike information criterion). Different letters indicate significant differences between sites. Values are expressed as mean ± SD.

**Fig 4:**
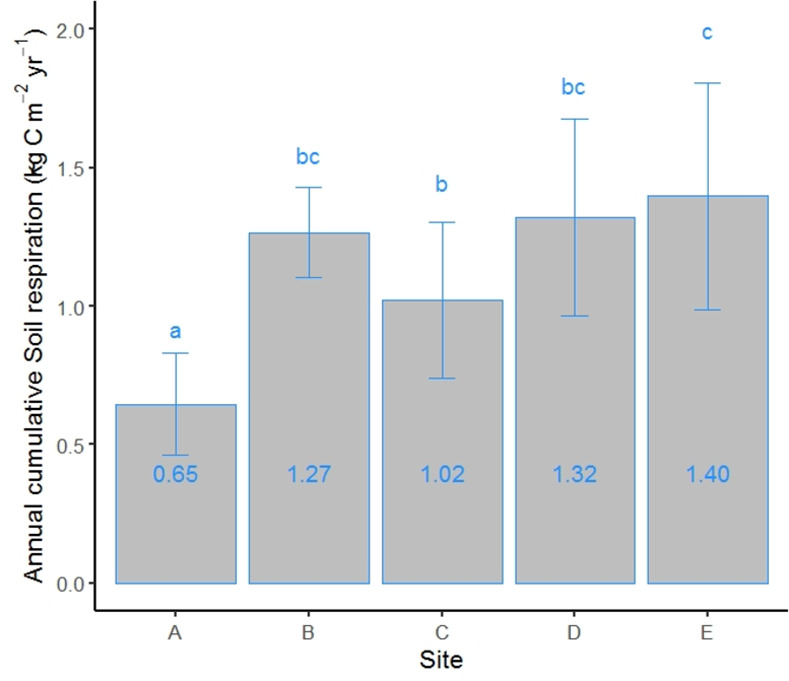
Total cumulative soil respiration (kg C m^-2^ yr^-1^) for the different sites. Error bars indicate standard deviation. Different letters indicate significant differences between sites.

The cumulative SR in site A was significantly lower than in the other sites (Fig 4). A nonlinear relationship between cumulative SR and elevation was also found (Table 2).

**Fig 5:**
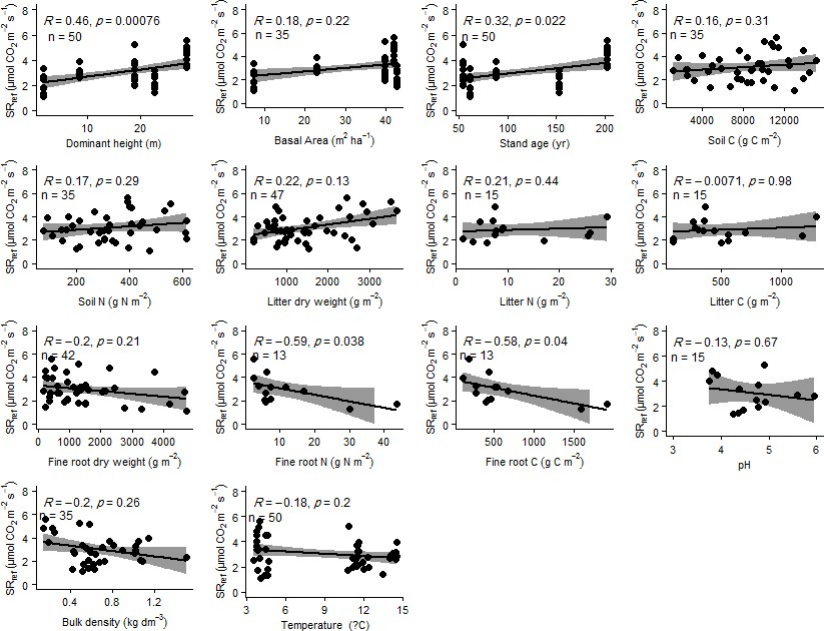
Relationships between reference soil respiration at 10°C (SR_ref_) and different site properties.

Soil organic C stock, mean dominant tree height, and litter dry weight best explained SR (SR_ref_, μmol CO_2_ m^–2^ s^–1^ at 10°C) in LMMs (VIF<10; Table 5). According to the model, about 16% of SR was explained by tree height (R^2^ = 0.16, Table 5). A positive association between SR and mean dominant tree height and stand age, and a negative association between SR and fine root C and N, were found by Spearman’s Correlation Test (Fig 5).

**Table 5:** Results of linear mixed-effects models testing the impact of biological variables on the SRref at 10°C.

Model variables	Value	VIF	p-value	R^2^
Intercept	1.51		0.004	0.29
SOC stock (kg C m^-2^)	< 0.01	1.20	0.67	< 0.01
**Dominant tree height (m)**	**0.05**	**1.21**	**0.01**	**0.16**
Litter dry weight (kg m^-2^)	< 0.01	1.05	0.09	0.07

Parameters in bold indicate significant correlations. VIF–Variance Inflation Factor.

## Discussion

### Organic C and N content along the altitudinal gradient

The recorded ranges of SOC and soil N stocks were 3891–10270 g C m^-2^ and 219–397 g N m^-2^ respectively (Fig 2). These values were within the ranges reported by other studies along elevation gradients [28, 64]. The lowest value was recorded at the lowest elevation, and our data corroborated an increase of the soil organic C stock with increasing elevation shown in other studies [9, 26, 27, 29, 64–67]. Being the main driver for the loss of soil organic C, low temperature can limit the decomposition of organic matter at high elevations. For this reason, elevation could induce a significant increase in SOC [25, 65, 67–69]. However, an increment in SOC stock was not linearly linked with elevation gradient and, therefore, our first hypothesis was not confirmed. The non-linear relationship of SOC stock with elevation and the high value of SOC stock in site C (with a mean temperature of 12°C) suggests that factors other than temperature have influenced SOC accumulation. Different microclimatic or micromorphological conditions caused by differences in slope angle and orientation can influence C storage in soils [35, 37, 70]. However, in the present study, all the sites were characterized by a similar slope and were all south or south-east facing; therefore, we exclude an influence of these factors on SOC accumulation. According to recent studies, SOC is not consistently related to variation in climatic conditions along elevation gradients, but is also strongly affected by productivity or by vegetation type/composition [32, 35, 71, 72]. Our data confirm a significant positive correlation of SOC stock with mean dominant tree height, basal area and stand age (Table 3). Generally, tall trees in a mature forest stand can increase litterfall production. Therefore, the higher amount of SOC found in the present study at intermediate elevation in the older stands (B and C) could be explained by higher site productivity, which is indicated by the mean dominant tree height and basal area [73, 74]. Standing leaf area and litterfall increase with tree size, although this relation is strongly species-dependent, with leaf area increasing much more in spruce than in Scotts pine forests.

Soil pH and bulk density are considered to be two of the main variables influencing soil properties other than SOC, soil microbial activity and soil respiration [75, 76]. Generally, at high elevation, higher precipitation and lower evapotranspiration rates reduce soil pH by increasing the leaching of basic cations [70, 77–80]. This was confirmed by the significant relationship between elevation and soil pH found in the present study (Table 2 and Fig 2). In addition, soil bulk density significantly diminished with increasing elevation (Fig 2 and Table 2). One of the main factors affecting soil bulk density is SOC content [81]. Therefore, the lowest values of soil bulk density at high elevation could be explained by the high stock of SOC, as confirmed by the negative association found between SOC stock and soil bulk density (Table 3), previously reported in other studies [9, 64, 67, 82].

### Factors affecting soil respiration

The total SR observed in our study sites is within the range reported for similar forests [28, 66]. The decrease in SR observed along the elevation increase could be explained by the reduction in temperature with elevation. The temperature was the main factor controlling and explaining most of the variability of SR, in agreement with other studies performed along altitudinal gradients reporting that temperature can explain between 55% and 76% of SR variability [8, 11, 16, 29, 68, 83]. Yet, the annual cumulative SR in site B was double that of site A, despite both sites experiencing the same mean annual temperature. This finding, together with nonlinear SR correlation with elevation and temperature, suggests that other environmental factors can have a role in regulating SR [36, 66]. For example, Grand et al. [38] reported that soil and vegetation heterogeneity strongly affect soil carbon efflux in complex geomorphic terrain. The five study sites were established on the same bedrock; therefore, the high SR rate in site B could not have resulted from a confounding effect of the soil parent material. Site B is an uneven-aged dense mature forest stand, with a mean dominant tree height of approximately 29 m (Table 1). Since tree height can be used as a proxy of GPP, the high SR rate in this site could be attributed to high GPP [11, 31, 71, 84–86], which can provide substrates for root and microbial respiration through photosynthesis [71, 87], supported by a significant positive relation between SR_ref_ and dominant tree height (Fig 5 and Table 5). Above- and below-ground tree size increases with age and, therefore, the growth of root biomass leads to higher SR as forest stands develop [39, 88–90]. In this context, our findings suggest that after removing the effect of temperature, productivity and tree size are principal factors affecting SR.

At a global scale, SR has been related to SOC stock, litter production and pH, and negatively correlated with soil bulk density; therefore, a high accumulation of SOC and litter could lead to an increase in soil respiration [22, 71, 91–93]. In the present study, the highest amount of SOC stock and dry litter weight were also observed in site B. However, we did not find a significant correlation between SR and SOC stock or litter dry weight (Fig 5 and Table 5).

An increase in SR has also been observed as a consequence of increasing soil pH between 4 and 7, because of the positive effect of pH on soil microbial activity within this range [1, 75, 94–96]. Conversely, SR and bulk density are generally negatively correlated, as a low SR indicates increasing rates of SOC accumulation and therefore a decrease in bulk density [92]. Furthermore, SR declines with increasing bulk density due to the lower soil porosity and oxygen availability for microbial activity in compacted soils [20, 65, 97]. However, our analysis did not confirm a significant correlation of SR with pH or bulk density (Table 5 and Fig 5). Prediction of SR is difficult because of a range of factors such as slope angle and orientation, and soil type [37, 38, 66, 70]. In the present study, by minimizing the confounding role of these parameters, we conclude that the most important factors controlling SR along an Alpine altitude gradient were temperature and dominant tree height. Therefore, a tall adult or mature forest stand with high productivity or GPP, and even more so a more complex and uneven-aged structured forest able to maintain high biomass levels over time such as site B, can significantly affect SR, suggesting that tree size and stand biomass are more relevant than stand age itself.

### Temperature sensitivity of soil respiration (Q10)

The temperature sensitivity of SR is an important ecological model parameter, and according to previous studies it is mainly controlled by temperature [16, 19, 98, 99]. The Q10 and SR_ref_ values found at site B (2.0 and 4.09, respectively) confirm those found at the same site by Acosta et al. [100]. Although at a smaller spatial scale, Acosta et al. [100] also found an increasing SR_ref_ to be a function of stand age (and consequently height). The significant trend of Q10 with elevation in our study confirms previous results, highlighting the higher sensitivity of high elevation ecosystems to global warming [16, 101–104]. However, the only significant difference was found between the Q10 value at site A (higher elevation) and the other sites (Table 4). The Q10 value in site A was also significantly higher than site B, which is characterized by a similar mean annual temperature. Previous studies have found that Q10 is negatively related to pH and positively related to SOC stock [61, 105]. However, in our study SOC stock and pH could not be the cause of the lower Q10 value found in site B which, compared with the other sites, is characterized by a lower pH value and a similar SOC stock content. The temperature sensitivity of SR can also be affected by forest structure [106]. Dense forest stands with a closed canopy can create a specific understory microclimate by providing a cool shelter during heat waves, which can decrease daily maximum air temperature by up to 5.1°C [107, 108]. Therefore, we can hypothesize that, since temperature range is positively linked with ecosystem respiration [109], the dense forest stand in site B, which has the highest dominant tree height, is less sensitive to global warming. This confirms what found by Niu et al. [110] in the same site and may explain the significant negative correlation between Q10 and mean dominant tree height (Table 3). Our study suggests that a close-to natural, continuous cover forestry system with a significant permanent presence of bigger individuals, particularly on sites with good forest productivity, can not only maintain current C stock in the biomass but also lead to a reduced sensitivity to temperature of SOC stock.

### Role of stand structural properties

While the correlation between forest productivity and respiration is well known [11], this research opens a new perspective regarding the possible link between stand structural parameters and temperature sensitivity of respiration in forest soils. In a mature forest several characteristics, differing from those of a younger forest, may influence soil respiration. While stand age is the most obvious, it does not appear to be tightly linked to soil respiration [39], and our research corroborates this. Forest stature can also affect forest soil microclimate; intact forests show a reduced temperature above the canopy [111, 112] and current studies are evaluating the impact of vegetation structural properties on soil temperature [113]. In addition, there is evidence of a positive trend between forest development and efficiency in the enhancement of soil C storage, most likely related to the presence of microbial communities and plant roots more efficient in SOC storage [114].

Among the other variables that may affect SR that should be evaluated there is the overstory LAI. A significant positive correlation between LAI and SR was reported by Migliavacca et al. [39]. This positive relationship can be explained by the relation between LAI and productivity. However, a high LAI can also intercept direct radiation, reducing thermal fluctuations at the soil level and hence SR sensitivity to temperature [102, 115]. The mature stand in site B has not only a high overstory LAI, but also a relevant presence of understory [116] able to intercept direct radiation passing through the uneven-aged canopy. The role of litter quality, and particularly of N content, is still debated. Long-standing leaves present in the mature stand (site B), which are depleted in N, may have (or not) a longer degradation time [117, 118]. However, only a more detailed experimental design would allow us to determine which of these different features of mature vs. young forest affect SR sensitivity to temperature.

## Conclusions

In this study, a significant nonlinear relationship between SR, SOC, Q10, and elevation was detected along the examined Alpine altitudinal gradient, rejecting our initial hypothesis of linearity. Our data confirmed a negative trend between SR and elevation, but a positive trend of SOC and Q10 with elevation. We, therefore, conclude that temperature is the major factor controlling annual SR, Q10, and SOC, but its regulating role may be strongly affected by site biological characteristics, particularly GPP or vegetation type/composition. The high Q10 value detected at high elevations confirmed a greater potential vulnerability of high mountain ecosystems to climate change, where small temperature changes can induce a stronger increase in CO_2_ emissions. However, the site with the tallest dominant height and more complex structure showed a high SRref and moderate Q10, indicating that maintaining a forest close to an old growth system, with a heterogenous and uneven-age structure, can reduce, to some extent, the effects of climate change on ecosystems and decrease the positive feedback due to soil CO_2_ emissions to the atmosphere.

## Supporting information

S1 File Forest inventory. (XLSX)

S2 File Soil respiration. (XLSX)

S3 File Soil and plant analysis. (XLSX)

S4 File Temperature data and modeling. (XLSX)

S5 File Equation parameters. (DOCX)

S6 File Soil_respiration and air temperature. (TIF)

S7 File Observed and predicted soil resp. (TIF)
