# Supplementary material for: Soil respiration variation along an altitudinal gradient in the Italian Alps: Disentangling forest structure and temperature effects
Source: PLoS One. 2021 Aug 17;16(8):e0247893. doi: 10.1371/journal.pone.0247893 (PMC8370607; doi:10.1371/journal.pone.0247893)
Supplement: S5 File — (DOCX) [file pone.0247893.s005.docx]

**S5. The developed equations by linear and polynomial regressions between the different parameters tested and elevation. BD – soil bulk density; Q10 – temperature sensitivity of soil respiration; SR- cumulated soil respiration; SR_ref_ – respiration at the 10°C reference temperature; Elev – elevation.**

| Parameters | Elevation | |
| --- | --- | --- |
|  | linear | Polynomial regression |
| Soil C ( g C m^-2^) | Soil C = 4227.46 +3.21 Elev | Soil C = -9.19 +0.1 Elev - 0.004 Elev^2^ |
| Soil N ( g N m^-2^) | Soil N = 214.51 +0.09 Elev | Soil N = 8.55 +0.35 Elev - 0.0001 Elev^2^ |
| SR (kg C m^-2^ yr^-1^) | Soil C = 1.58 - 0.00035 Elev | Soil C = 1.33+0.00017 Elev - 0.0000002 Elev^2^ |
| Q10 | Q10 = 1.34 + 0.0005 Elev | Q10 = 2.13 - 0.001 Elev + 0.0000006 Elev^2^ |
| SR_ref_ (kg C m^-2^ yr^-1^) | SR_ref_ = 3.13 - 0.00009 Elev | SR_ref_ = 2.26 +0.0017 Elev + 0.0000007 Elev^2^ |
| Fine root mass (g m^-2^) | Fine root mass = 674.0 +0.62 Elev | Fine root mass = 2.24 + 2.76Elev + 0.0014 Elev^2^ |
| Root C (g C m^-2^) | Root C = -17.10 +0.52 Elev | Root C = 1.16 – 1.94 Elev + 0.001 Elev^2^ |
| Root N (g N m^-2^) | Root N = -0.65 +0.01 Elev | Root N = 2.97 -0.05 Elev - 0.00003 Elev^2^ |
| Litter mass (g m^-2^) | Litter mass = 1040.26 +0.33 Elev | Litter mass = 1.91 +2.10 Elev - 0.0007 Elev^2^ |
| Litter C (g C m^-2^) | Litter C = 438.23 +0.04 Elev | Litter C = 1.3+0.001 Elev - 0.0007 Elev^2^ |
| Litter N (g N m^-2^) | Litter N = 1.03 +0.0008 Elev | Litter N = 1.3+0.001 Elev - 0.0001 Elev^2^ |
| pH | pH = 5.80 - 0.0008 Elev | pH = 7.08 - 0.0035 Elev - 0.000001 Elev^2^ |
| BD (kg dm^-3^) | BD = 1.06 - 0.0003 Elev | BD = 1.18 +0.00054 Elev - 0.00000009 Elev^2^ |
