# Supplementary figures and images for: Soil respiration variation along an altitudinal gradient in the Italian Alps: Disentangling forest structure and temperature effects
Source: PLoS One. 2021 Aug 17;16(8):e0247893. doi: 10.1371/journal.pone.0247893 (PMC8370607; doi:10.1371/journal.pone.0247893)

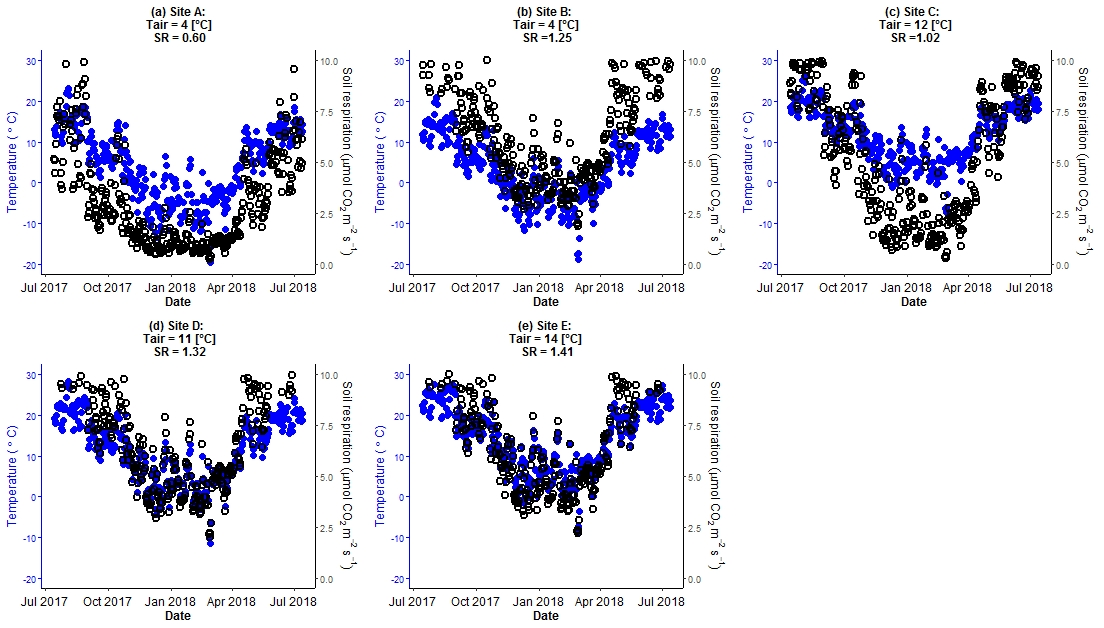

Supplement: S6 File — (TIF) [file pone.0247893.s006.tif]

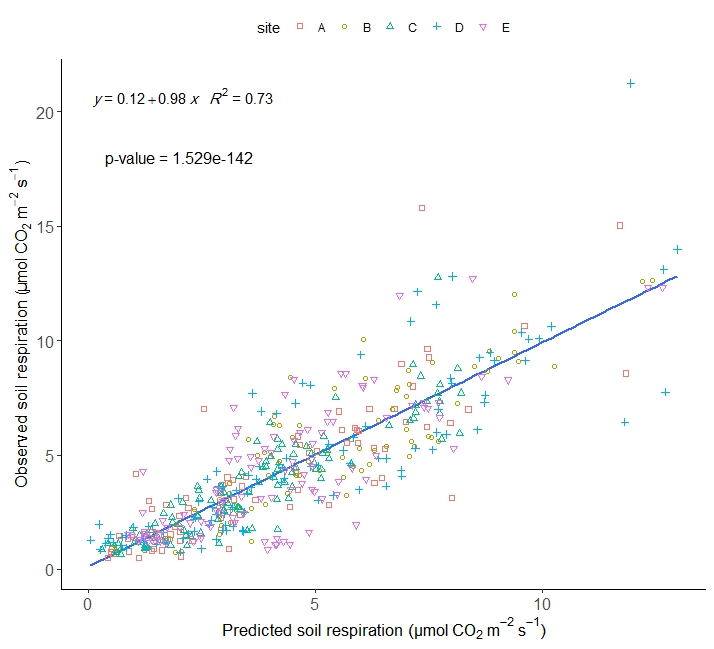

Supplement: S7 File — (TIF) [file pone.0247893.s007.tif]
